# COVID-19 affected elite track-and-field athletes’ Olympic preparation before Tokyo 2020 compared to Rio 2016

**DOI:** 10.1038/s41598-025-86883-2

**Published:** 2025-02-19

**Authors:** Alexander Banning, Edda van Meurs, Dennis Dreiskämper

**Affiliations:** 1 Department of Sport & Exercise Psychology, Institute of Sport and Exercise Sciences, University of Münster, Horstmarer Landweg 62b, D-48149 Münster , Germany; 2 Development and Learning (Sports Psychology), Department of Sport and Sport Science, TU Dortmund University, Dortmund, Germany

**Keywords:** Track and field, Performance progression, Corona, Olympics, Random effects model, Human behaviour, Statistics, Developmental biology

## Abstract

The COVID-19 pandemic has had a significant impact on elite sport by postponing the Olympic Games Tokyo 2020 four months before the original start. This impacted athletes’ macro-cycle periodization, psychological stressors and resources. We analyse whether track-and-field athletes were able to maintain their performance levels successfully across the last two Olympic cycles, controlling for age, gender and doping prevalence. For this, worldwide competition results (excluding multi-events & relays) of at least national level since London 2012 and up to Tokyo 2020 were retrieved. Individual performance curves were analysed using hierarchical multilevel modelling. Individual baselines (random intercept) and developments (random slope) were analysed. 2,383 athletes (52% male) recorded 15,766 outcomes since London 2012. The final conditional growth model (ICC = 48%) shows that performances increased in the wake of Olympic games, dropped significantly in 2020 and recovered beyond previous form in 2021. There was no significant difference between men’s and women’s developments. Age was a significant predictor (*b* = 0.17, *SE* = 0.02), but doping violations was not (*b* = 0.01, *SE* = 0.03). These results showcase performance trends in international athletics and their variability, present an overall successful periodization to achieve peak performance at Tokyo 2020, and discuss predictions for track and field at Paris 2024.

## Introduction

In 2021, in the wake of and during the Olympic Games in Tokyo (Tokyo 2020), 12 athletic world records (WRs) were broken: in men’s 60 m hurdles, men’s and women’s 400 m hurdles, 1,500 m and 10,000 m running, triple jump, 4 × 100 m swimming relay, 100 m swimming butterfly and weightlifting. This is equal to the number of WRs in track and field in 2012 and 2016 together, when track-and-field athletes were at their peak performance for the Olympic Games London 2012 and Rio 2016. This is surprising for two reasons: (1) the increasing difficulty of breaking WRs, and (2) the COVID-19 pandemic.

First, it is assumed that WRs become more and more difficult to improve because performances draw closer and closer to what is humanly possible^1^. Looking at the frequency and relative improvement of WRs, Berthelot et al.^1^ reported that WRs in athletics and swimming are broken less frequently with every decade, and if they are broken, the relative improvements become smaller and smaller. Their archival data show that instead of a linear increase of overall performance across athletes, the pattern follows a logarithmic trend, indicating that these sports seem to draw closer to their natural peak (cf. p. 3). Fluctuations within this trend are explained by historical, technological, pharmacological and physiological factors. For example, Berthelot et al.^1 ^highlight the negative impact of world wars and the chances of technological advances. In an archival analysis, the 100 m sprint was influenced the least by technological innovations over the last century^2^. One of the most prominent prevalences occurs in short and long distance running in track and field. Here, WADA test results have found between 58 and 159 cases through Adverse Analytical Findings (AAF) and A-Typical Findings (ATF)^3–9^. AAF refers to the identification of a prohibited substance shown by a positive test result, whereas ATF is defined as an inconclusive finding that does not directly lead to a violation of the WADA list but requires further investigation. The vicious circle of implementing new doping methods and substances and the ability of WADA and NADOs to identify them is discussed intensively in the literature (for an overview, see^10^). This counterplay between inventing new equipment/supplements (performance increase) and new rules that ban these inventions (performance decrease^11^) contributes to the fluctuations. However, these general assumptions about the prevalence in single disciplines are hardly usable to understand individual performance development (despite those being convicted). Lastly, the plateau across athletes speaks to the physiological limitations of human performance, like the age-determined performance stagnation^12,13^.

Second, the WRs of 2021 occurred after a year of lockdown, characterized by reduced training and competition possibilities and psychological stressors due to the COVID-19 pandemic. COVID-19 was declared a pandemic by the WHO on March 11^th^, 2020. To prevent the worldwide spread, governments across the world enforced restrictions on travel, leaving home and social contacts, with local differences^14^. With some exceptions, these restrictions also applied to sports in general and elite athletes in particular, who, for example, prepared for the Olympic Games in Tokyo 2020^15^.

The COVID-19 pandemic led to multiple challenges unique to elite sports such as the systemic restrictions by governments and the postponement of Tokyo 2020. The most obvious impact of the delay of the Olympic Games in Tokyo from 2020 to 2021, which was declared in April 2020, is the disruption of periodized training. Periodization is the “purposeful sequencing of different training units […] so that athletes could attain the desired state and planned results”^16^, that is, peak performance at a specific date or competition^17,18 ^by applying periodic cycles in line with the competition schedule of the athlete. At the highest level of periodic cycles is the multi-year preparation, where the Olympic quadrennial cycle is of particular importance^16,19^. Accordingly, the delay of Tokyo 2020 effectively meant that the Olympic athletes’ multi-year cycle was stretched from four to five years, which led to a delay of the maximum peak performance. This in turn meant that coaches and athletes returned to general preparation or transition periods to start a retraining process^20,21 ^and to resume specialized training before the new season’s highlight to maximize performance beyond the estimated peak in 2020. Early studies reported decreased workloads in the beginning to adapt to the training restrictions and prevent injuries^22–24^, a recovery to previous levels one month after the lockdown and a drop again after six months, coinciding with second lockdowns and restrictions in many countries^24,25^. On the micro-level or day-by-day training, elite athletes experienced impactful restrictions: they had to train on their own, received remote coaching^26^, were not allowed to use sport facilities and did not receive the usual medical care^27^. The lack of resources and facilities led to fewer training sessions, different foci of each session and reduced load according to self-reports^26^.

The delay of the Games did not only influence the athletes’ periodization, but also their motivation, psychological stress level and mental health. Once the delay had been announced, athletes experienced symptoms of burnout, feeling of alienation and poor coping^28^. Schinke et al.^28^reported that some elite athletes formed constructive responses and gained intrinsic motivation to cope with the strenuous circumstances. A literature review of 16 studies found that the pandemic increased athletes’ psychological distress, anxiety, and stress in general^29^. They highlighted the importance of athletes’ resilience and personality to cope with these challenges and defined them as mediating factors for the stressors^30^. Moreover, female athletes and those competing at higher levels of play were at higher risk for a pandemic-related decrease in mental health^31^. Some athletes were able to cope by introducing new methods of home training programs and quarantine training camps^31^. Since studies prior to the pandemic have already described the impact of high stress and poor mental health on elite athletic performance and injury risk, it is expected that this mediates the effect of the pandemic on objective sporting performances^23^.

The pandemic introduced uncertainty, unpredictability, and uncontrollability for everyone. The resulting increased anxiety^32,33^, poor sleep quality^34^, depressive symptoms^35^, dissociation from the athlete’s identity^35^, and decrease of intrinsic motivation^36^, have all been found to be detrimental to performance. Because of this, the pandemic was called a changing event in an athlete’s sporting career due to its distressing and derailing nature^35^. Factors that usually help athletes to cope with stressful adaptations and to build resilience (e.g., perceived social support, sense of belonging/meaning, motivational environment, daily routines), were often restricted or prohibited by COVID-19 regulations^35,37^.

Considering these changes and stressors due to the COVID-19 pandemic, the question is raised how individual athlete’s performance developed in the two challenging years 2020 and 2021. Beyond pandemic-related retrospection, it is important to note that studying the effects of the pandemic provides insights into how performance and well-being are affected by disruptions to training schedules, competition calendars, and access to resources. While pandemics may be rare, similar disruptions can arise from injuries, congested competition schedules, or geopolitical events^38^, all of which create unforeseen breaks or challenges in preparation. Analyzing individual progressions and responses to these changes during the pandemic can help inform training protocols and resilience-building approaches for athletes facing comparable disruptions in the future. Moreover, this research highlights the importance of psychological support and flexible periodization. Track-and-field offers a particularly clean operationalization of performance development across Olympic cycles, as their seasonal bests are objective representations of their performance capacities. To investigate the influence of the postponement of Tokyo 2020 on track-and-field athletes’ performance development, this study analyzes and compares each Olympic athlete’s seasonal bests (SB) across the Olympic cycles Rio 2016 and Tokyo 2020 and quantifies the effects of a five-year cycle compared to a four-year, while controlling for age, sex differences, and doping affinity. We hypothesize that individual SB depend on the time in the Olympic cycle (*H1*): the average SB will increase with every year until the Olympic Games. This development is assumed to be linear (*H1.2*). This also predicts that the overall improvement over the five-year Tokyo 2020 cycle should be larger than the overall improvement over the four-year Rio 2016 cycle. This would explain the large number of world records in the season 2021. Moreover, we control for the linear (*H2.1*) and quadratic (*H2.2*) effect of age on performance. We assume that age predicts performance positively, until it plateaus at a certain age. We expect the development over the seasons to be stable for both sexes (i.e., there is no interaction between the year of competition and sex of the athlete, *H3*). We further hypothesize that the SB of individual athletes are predicted by the doping affinity of each discipline category within track and field (*H4*), indicating that a higher doping affinity of the discipline category is associated with better performance developments.

## Methods

All analyses were conducted in RStudio version 4.3.2^39^. A subsample of the extracted records, a list of all included competitions and the script for replication can be found in this OSF repository: https://osf.io/2evxh/.

The research question was answered using performance records from World Athletics from the end of the season 2012 until the end of the season 2021. World Athletics is the international governing body for athletics that coordinates the competitions around the globe. Since 2018, these competitions are organized in ten different categories depending on the level and significance of the competition (https://worldathletics.org/world-ranking-rules/basics^40^). The category “OW” reflects the strongest competitions (e.g., World Championships, Olympic Games), followed by Diamond League Finals (DF), Gold World Athletics competitions and equivalents (GW) and Area Senior Outdoor Championships (GL). Other competitions and meetings are classified in A to F. An overview of the included competitions, athletes and performances per competition category can be found in Supplement 3. Athletics splits into track events, field events, cross-country, mountain, road and ultra-running as well as race walking. The present analysis focuses on the track-and-field events, covering 18 disciplines for both genders. Furthermore, the track and field season is split into an indoor and an outdoor season. The latter starts around March every year and ends around October. It follows the same rules and regulations of athletics competitions at the Olympic Games. We exclude the indoor season because not all Olympic disciplines are contested indoors, and because conditions have been shown to be different indoors for some disciplines, distorting performance estimations^41,42^.

### Data

Data was scraped from www.worldathletics.org/competition/ on March 16^th^, 2022, where the end-table performance of every athlete at each selected competition is documented. Most events and competition results under the new rules and regulations of World Athletics are documented since 2018. Archival data prior to 2018 is scarce, but major competitions are available. As this paper aims to assess the predictability of the Olympic track-and-field disciplines from ratified performances, the selection of competitions was limited to outdoor events. Road running and indoor-competition results were not considered. We included all major event series of World Athletics (World Athletics Championships, World Athletics Continental Cup, Diamond League, World Athletics Challenge/World Athletics Continental Tour, Olympic Games, cf. https://www.worldathletics.org/competition), area and national championships, and renown meets since the first day after the London Olympic Games 2012 (August 3^rd^, 2012) until the last day of the Tokyo Olympic Games 2020 (August 8^th^, 2021).

For all performance records, data was extracted using the R-package *rvest*^43^. The data included information on the event (name, category, date, location/host nation), the competition (discipline, round), the athlete (full name, birthday, nationality, gender as indicated by the competition group), individual performances (time run or furthest distance thrown/jumped, converted into points for the multi-events, place), and wind (in m/s). Based on the competition date, events were assigned to the Rio Olympic Games 2016 cycle (August 3^rd^, 2012 to August 20^th^, 2016), Tokyo Olympic Games 2020 cycle (August 21^st^, 2016 to August 8^th^, 2020) and the additional COVID-19 year 2021 (August 8^th^, 2020 to August 8^th^, 2021). Missing data (e.g., competition information, birth days) was manually researched and observations were excluded if it could not be retrieved (*N* = 6 observations).

To analyze performance developments across Olympic cycles, we included only those athletes who competed at either Rio 2016 or Tokyo 2020. Moreover, several competition results were excluded: if no measurement took place (NM), if the athlete did not start (DNS) or finish (DNF), or if the athlete was disqualified (DQ). Multi-events (heptathlon, decathlon) and relays were excluded since performance development of these combined events are not independent and are not operationalizations of individual capacities. Additionally, outliers were excluded from further analysis as a function of age and extreme deviation from the average performance per discipline and gender (± 3*SD* from *M*). Additional checks like controlling for name changes (e.g., due to marriage) were included. The analysis was aimed at personal developments rather than winning or losing or tactical running, hence, seasonal bests (SB) per discipline per year were calculated for each athlete.

Doping affinity of each discipline was controlled for with official WADA results. Doping affinity was operationalized as the prevalence for cases per gender and discipline in each year. Disciplines were merged into five categories, i.e., short distance run (up to 400 m), middle distance run (up to 1,500 m), long distance run (3,000 m and more), throwing competitions (discus, javelin, hammer, shot put), and jumps (high jump, long jump, triple jump, pole vault). Data was retrieved from the official Anti-Doping testing Fig. 2^3–9^. Only urine testing figures were used, as others (such as blood testing) were not available for all included years. The number of tests in competitions (IC) and out of competitions (OOC) are documented separately for each year and summed up. The average percentage of positive tests per year and discipline category is presented in Table 1. Irregular findings (i.e., positive test results) are summarized from AAF and ATF prevalences. The score of doping affinity per year and discipline category (in %) was calculated as the sum of positive AAF and ATF divided by the sum of positive and negative AAF and ATF.

**Table 1:** Sample descriptive statistics across all seasons. The table provides the number of individual athletes who competed per year as well as how many competitions and how many performances were included in the present analysis. The average age and standard deviation as well as the average number of positive doping tests per discipline category (short, middle, long distance, jumping, throwing) is documented.

**Year**	**Rio 2016**					**Tokyo 2020**					**Total**
	**2012**	**2013**	**2014**	**2015**	**2016**	**2017**	**2018**	**2019**	**2020**	**2021**	
*Competitions*	5	21	22	22	19	23	197	334	164	260	809
*Performances*	209	912	950	1,234	1,743	1,240	1,940	1,969	1,035	1,802	13,034
*Athletes* *(% female)*	207 (53.14%)	890 (49.1%)	934 (49.36%)	1,213 (48.23%)	1,715 (48.75%)	1,218 (48.93%)	1,832 (47.27%)	1,838 (47.44%)	971 (48.20%)	1,701 (47.56%)	2,386 (51.80%)
Nations	62	130	109	145	179	153	169	184	112	192	200
Age *M* (*SD*)	24.82 (3.92)	23.32 (4.16)	23.79 (4.41)	24.62 (4.4)	25.44 (4.54)	25.15 (4.34)	25.6 (4.34)	26.03 (4.28)	27.03 (4.21)	27.64 (4.28)	25.66 (4.49)
*Doping*				1.40% 132/9,445	1.51% 297/19,691	1.07% 258/24,174	0.97% 270/27,751	1.00% 306/30,587	0.87% 141/16,242	0.8% 217/27,029	1.05% 1,621/154,919
Short distance				1.6% 38/2,370	1.63% 85/5,230	1.18% 72/6,113	0.86% 55/6,400	0.81% 60/7,372	1.14% 48/4,201	1.05% 79/7,491	1.12% 437/39,177
Middle distance				0.82% 9/1,096	0.96% 21/2,197	0.54% 14/2,595	0.98% 30/3,073	1.03% 34/3,294	0.44% 8/1,839	0.73% 21/2,865	0.81% 137/16,959
Long distance				1.59% 55/3,463	1.62% 109/6,716	1.29% 114/8,867	1.17% 133/11,411	1.15% 142/12,335	1.11% 64/5,779	0.73% 68/9,359	1.18% 685/57,930
Throws				1.23% 16/1,306	1.59% 46/2,896	1.23% 44/3,582	0.95% 34/3,582	0.98% 39/3,964	0.54% 12/2,224	0.84% 32/3,799	1.04% 223/21,353
Jumps				1.16% 14/1,210	1.36% 36/2,652	0.46% 14/3,017	0.55% 18/3,285	0.86% 31/3,622	0.41% 9/2,199	0.48% 17/3,515	0.71% 139/19,500
*World records*	4	0	2	2	6	0	2	7	7	10	40
by *n* athletes	4	0	2	2	5	0	2	6	4	8	33

### Analysis

Athlete development was modeled using each athlete’s SB derived from the available competition data for all individual athletes who competed either at Rio 2016 or Tokyo 2020. Since most athletes competed at the highest international level for more than one year, performances were nested within individual athletes and were the basic unit of analysis. To account for this nested structure and interdependence between observations, multilevel modeling was applied using the *lme4-package*^44^. Multilevel models, also known as mixed-effects models or growth models, are extended forms of regression analyses to account for nested structures or clusters within the data^45,46^ while allowing to control for additional confounding factors. They model the error term as clustered within the nesting unit (here: athlete) to avoid an interdependence of the error terms by modeling the random deviations in the relationship between the predictors and the outcome for each cluster of data. In multilevel models, data is modeled within the level-2 clusters:

$${Y}_{ij}={\beta }_{0i}+{e}_{ij}$$

where ${Y}_{ij}$ is the outcome variable of performance $i$ by athlete $j$, ${\beta }_{0i}$ the mean outcome score of each athlete, and ${e}_{ij}$ the within-cluster error term reflecting the deviation of performance $i$ by athlete $j$ from their mean, ${\beta }_{0i}$. Here, the number of random clusters is large (*k*= 2,383 athletes). Hence, mixed-effects modeling was applied^47^ to build a 2-level growth model and account for the cluster affiliation (expressed by the explained variance on level 2 as the intraclass correlation coefficient ICC). In other words, the SB per year (level 1) are nested in the individual athletes (level 2).

Performance development was modeled as SB (mean centered per discipline and gender) over time (centered to 2013 as intercept, because the reliability of 2012 as an intercept is compromised due to missing competition documentation) while adjusting for age (mean centered), gender of the athlete (level-2 predictors), and doping affinity per discipline and season (level-1 predictor). Model fit was assessed by AIC and −2LogL deviation statistic^48^, which express how well the model and the variance estimate fit the available data. The significance test of the −2LogL deviation indicates whether the model fits the data significantly better than a model for comparison (here: the previous model). In addition, we assessed the model’s validity using the ICC and the coefficient of determination (COD) or conditional *R*^2^. Conditional *R*^2 ^describes the variance explained by the entire model. It is derived as a function of the variance of the fixed-effect component, the variance of the random effects, and variance at the observation level^49^, which was calculated from the delta method. We used hierarchical modeling with maximum log-likelihood estimation by adding each predictor stepwise until the model fit AIC and −2LogL deviation statistic did not improve any further. This was done in the following order: unconditional means model (model 0), fixed slope model comparing all seasons to 2013 with random intercepts (model 1), as well as modeling time as a linear fixed slope (model 1.1), adding age as a control variable (model 2.1), adding age as a power function to allow for the non-linear changes in performance progression (model 2.2), adding gender as an interaction with time (model 3), and adding doping affinity (model 4). With all predictors included, the overall model reads:

$$performanc{e}_{ij}={\beta }_{0j}+{\beta }_{1j}Yea{r}_{ij}*gende{r}_{j}+{\beta }_{2j}ag{e}_{ij}+{\beta }_{3j}dopin{g}_{ij}+{e}_{ij}$$

where $Yea{r}_{ij}$ is interacting with ${gender}_{j}$ while controlling for $ag{e}_{j}$ and ${doping affinity}_{ij}$. Here, ${gender}_{j}$ and $ag{e}_{j}$ are level-2 predictors acting at the within-athlete level to explain the between-cluster of athlete-level variance of the data. The multilevel models were built using the *lme4*-package^44^. The corresponding ICC was calculated with the *performance*-package^50^. We report unstandardized effect sizes, which is in line with general recommendation^51^. We add Cohen’s *d*, which is derived from *b* by the *EMAtools*-package^52^. Cohen’s *d* is interpreted as small (*d* < 0.1), medium (*d* < 0.3) or large (*d*> 0.3)^53^.

## Results

### Descriptives

Since the London Olympic Games 2012, worldathletics.com has documented the results of *N* = 2,650 ratified track-and-field outdoor events, where 2,383 Olympic athletes competed. 185 SB were excluded from the descriptive statistics and analyses as outliers (± 3*SD* from *M*). Not all data was publicly available – detailed documentation exists only for all competitions since 2018. Table 1 shows that the number of documented SB increased with the years, which in turn increases the reliability of analyses for later years and, more specifically, the second Olympic cycle. Compared to the previous year, international track-and-field activities decreased in 2020, because many events were restricted: less competitions were held in 2020 compared to 2019 (∆ 2019–2020: 170, 51%), fewer athletes participated (∆ 2019–2020: 867, 47%) and less doping tests were conducted (∆ 2019–2020: 14,345, 47%). The number of world records increased throughout the Olympic cycle before Rio 2016, dropped in the following year and increased again in the wake of Tokyo 2020 (Table 1). In Supplement 1 and 2, the annual performance progression per discipline is displayed as averages of all Olympic athletes’ SB.

It is not only the number of competitions that decreased in 2020 due to the COVID-19 pandemic. A total of 234 athletes did not compete in 2020 and 2021, although they had competed in 2019 and 93 had competed regularly since 2017. The athletes who did not compete after 2019 anymore, were characterized by their relatively older age (*M* = 28.69, *SD* = 4.21, *t*(305.90) = −9.35, *p* < 0.001) and their relatively lower scaled performance (*M* = −0.51, *SD* = 1.06, *t*(281.50) = 8.39, *p* < 0.001) compared to the athletes who continued throughout and after the pandemic.

### Main analysis

The main analysis operationalizes performance as the SB per year and discipline of the athletes in a 2-level growth model. Table 2.and Table 3. present the results of the stepwise modeling process. The ICC of the unconditional means model (Model 0) indicate that of the total variance in performance, 47.1% was attributable to between-athlete variation and 52.9% was attributable to within-athlete variation. The unconditional means model was built with *N* = 15,766 observations (*k* = 2,383 athletes). By accounting for the clusters in the data, the scaled average performance was *b*_0_ = −0.09, 95% CI [−0.12; −0.06].

**Table 2.** Fixed effects of hierarchical multilevel model building. The predictor age was mean-centered, the predictor doping affinity is standardized (*M* = 0, *SD* = 1). The Standard Error (SE) of the model coefficients is provided for each estimate to indicate the precision of the predictor coefficient. The *p*-value is derived from the *t*-statistic as part of the *lme4*-package.

**Fixed effects**	**Model 1**		**Model 2.1**		**Model 2.2**		**Model 3**				**Model 4**			
	*b*	*SE*	*b*	*SE*	*b*	*SE*	*b*	*SE*	*b*	*SE*	*b*	*SE*	*b*	*SE*
Season							*female*		*male*		*female*		*male*	
2012	0.10	0.06	0.12*	0.06	0.11	0.06	−0.09	0.08	0.41***	0.11				
2013 (Intr)	−0.36***	0.03	−0.22***	0.03	−0.33***	0.03	−0.33***	0.04	< 0.01	0.06				
2014	0.05	0.03	0.02	0.03	0.05	0.03	0.01	0.05	0.07	0.07				
2015	0.24***	0.03	0.18***	0.03	0.23***	0.03	0.21***	0.04	0.04	0.06	−0.04	0.04	0.04	0.05
2016	0.30***	0.03	0.20***	0.03	0.28***	0.03	0.30***	0.04	−0.03	0.06	0.07	0.04	−0.07	0.05
2017	0.25***	0.03	0.13***	0.03	0.23***	0.03	0.23***	0.05	< 0.01	0.06	−0.05	0.04	−0.04	0.06
2018	0.23***	0.03	0.08*	0.03	0.21***	0.03	0.20***	0.04	0.01	0.06	−0.10**	0.04	−0.04	0.05
2019	0.33***	0.03	0.15***	0.03	0.30***	0.03	0.26***	0.04	0.08	0.06	−0.06	0.04	0.03	0.05
2020	0.17***	0.03	−0.04	0.04	0.14***	0.04	0.11*	0.05	0.06	0.07	−0.25***	0.05	0.01	0.06
2021	0.51***	0.03	0.27***	0.04	0.47***	0.04	0.50***	0.05	−0.07	0.06	0.15***	0.04	−0.13*	0.05
Age			0.03***	0.00			< 0.01	0.00			0.02***	0.00		
Age^2^					< 0.01	0.00								
Doping %											−0.02	0.01		

Note: *p < 0.05, ** *p* < 0.01, *** *p* < 0.001.

**Table 3.** Random effects and model fit of hierarchical multilevel model building. σ2, τ00, and the ICC describe the random variability introduced by the data clustering, while the Log-Likelihood parameter describes the model fitted using maximum likelihood.

	Model 1	Model 2.1	Model 2.2	Model 3	Model 4
Random effects					
σ^2^	0.52	0.52	0.52	0.52	0.56
τ_00_	0.52	0.52	0.52	0.52	0.56
ICC_conditional_	48.41	47.09	48.1	48.11	39.58
*N*	15,766	15,766	15,766	15,766	4,495
*k*	2,383	2,383	2,383	2,383	868
Model fit (logL)	−19,519.88	−19,465.74***	−19,518.22	−19,502.06***	−5,712.11

Note: If logL *** → model fit significantly better than previous model.

**Fig. 1:**
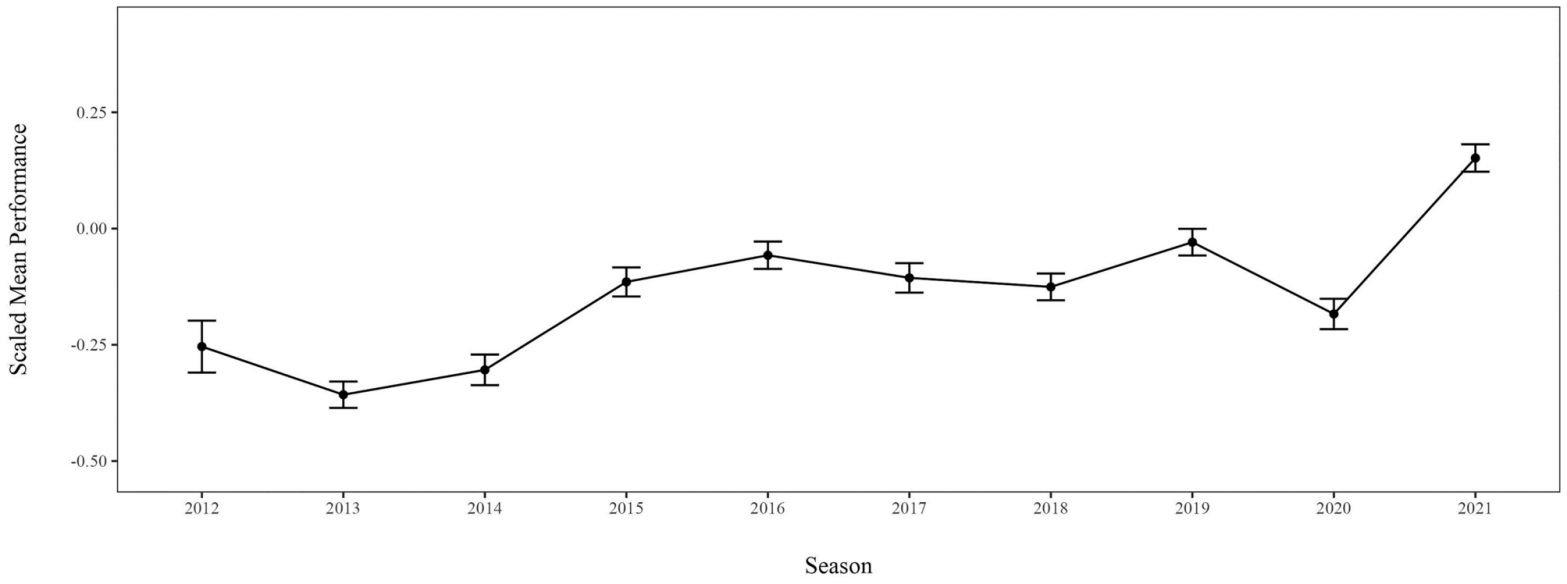
Visualization of the Estimated Means (*EM*) and Standard Errors (*SE*) derived from Model 1 with the predictor season. The scaled performances across all disciplines increase within each Olympic cycle (Rio 2016: 2013–2016; Tokyo 2020: 2017–2021) and peak in the Olympic year (2016; 2021). A visible decrease occurred in 2020 during the COVID-19 pandemic.

Throughout the stepwise modeling process (Model 1–4), the ICC remained stable between 46%−48%, justifying the choice of a mixed-effects modeling (see Table 3.). The *b*-estimations in Table 2. represent the average scaled increase or decrease of SB in comparison to the baseline/ intercept, here season 2013. The first conditional model analyzed the differences in performances over the nine seasons, which improved the model fit, −2LL_model 1_ = −19,519.88, χ^2^(12) = 477.85, *p* < 0.001, τ = 0.72, ICC = 48%, *R*^2^_cond._ = 0.50. The results (for an overview, see Fig. 1) indicate that averaging over the disciplines, performances decreased after the Olympic Games in 2012 to the lowest level across all seasons (decrease from 2012 to 2013: *b* = −0.09 [−0.20; 0.01], *d* = −0.03), and from there increased again (e.g., increase from 2013 to 2016: *b* = 0.30 [0.24; 0.36], *d* = 0.18). Similarly, after Rio 2016, SB decreased again slightly (from 2016 to 2017: −0.050 [−0.052; −0.048]) and increased by *b* = 0.262 [0.260; 0.264] from 2017 to 2021. Most noticeably, the average performance during 2020 was not in line with the previous or the following year: While scaled SB in 2019 was *b*_2019_ = −0.03 [−0.09; 0.02], and 2021 was *b*_2021_ = 0.15 [0.10; 0.21], in-between, performances dropped to *b*_2020_ = −0.19 [−0.25; −0.13]. The intercept, that is, the average performance in 2013 (*b*_0_ = −0.36 [−0.41; −0.30]), is significantly below the overall average of all observations (*SE* = 0.03, *p* < 0.001), which supports the hypothesis that track-and-field performances increased since then. Seasons 2012 (*p* = 0.087) and 2014 (*p* = 0.156) are the only seasons that do not differ significantly from the intercept (see Table 2.).

When modeling this development linearly across both Olympic cycles (Rio 2016, Tokyo 2020), the model fit decreased significantly, −2LL_model 1.1_ = −19,596.82, χ^2^(6) = 153.89, *p* < 0.001, τ = 0.73, ICC = 48%, *R*^2^_cond._ = 0.49. There appears to be a significant linear increase over the four years until the Olympic Games in 2016, *b* = 0.10 [0.08; 0.11], *p* < 0.001, *d* = 0.20, and a smaller linear increase over the five years until the Olympic Games in 2021, *b* = 0.06 [0.04; 0.08], *p* < 0.001, *d* = 0.13. Hence, while the linear interaction was found to be significant, it did not explain the variance in individual SB better than modeling the years non-linearly.

**Fig. 2:**
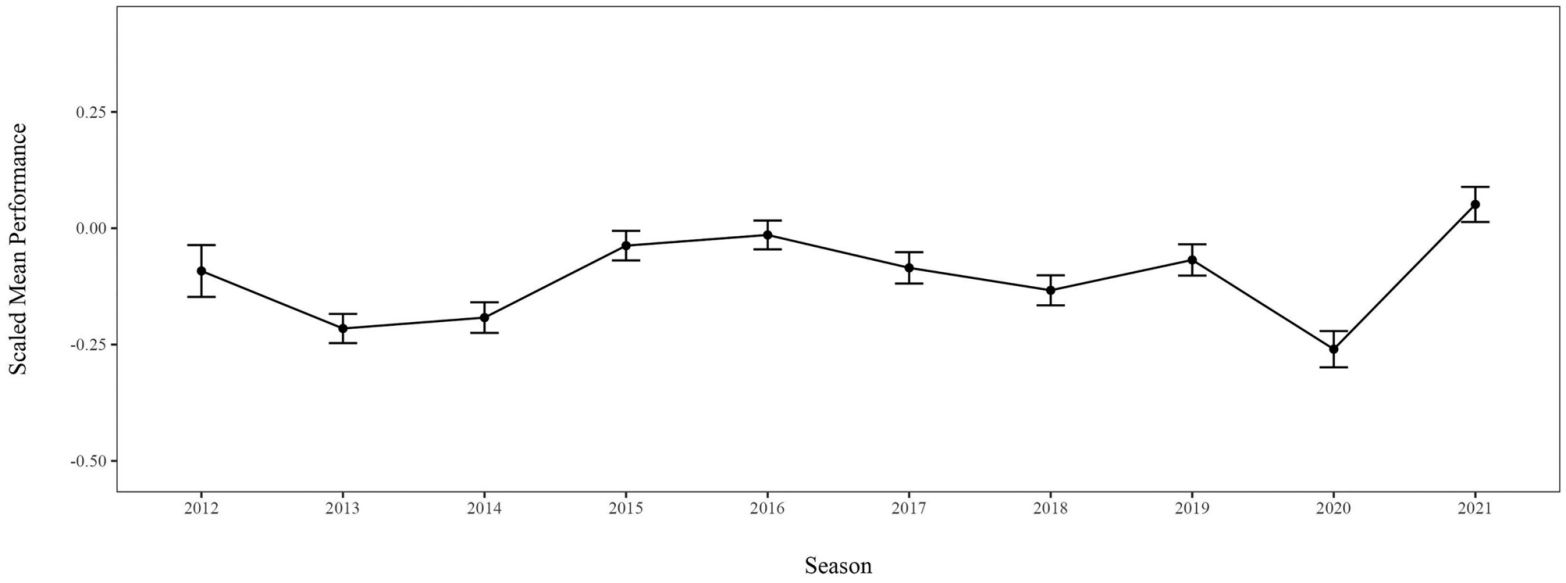
Visualization of the Estimated Means (*EM*) and Standard Errors (*SE*) derived from Model 2.1 with the predictors season and age. Again, the scaled performances across all disciplines increase within each Olympic cycle (Rio 2016: 2013–2016; Tokyo 2020: 2017–2021) and peak in the Olympic year (2016; 2021). The performance decrease in 2020 persisted. Similar results were found when age^2^ was used as a predictor.

Adding age as a level-2 predictor again improved the model fit, −2LL_model 2.1_ = −19,465.74, χ^2^(13) = 108.28, *p* < 0.001, τ = 0.72, ICC = 47%, *R*^2^_cond._ = 0.51. Performance increased with age, *b* = 0.16 [0.13; 0.19], *p* < 0.001, *d* = 0.18, and accounted for enough variance in the data to cause some changes in the estimators of the yearly performance averages (see Fig. 2). Table 1 shows that the average age decreased in the season after Rio 2016. Modeling age as a power function (age^2^) did not improve model fit, −2LL_model 2.2_ = −19,518.22, τ = 0.72, ICC = 48%, *R*^2^_cond._ = 0.50, hence, the less complex predictor from model 2.1 is kept for further analyses.

**Fig. 3:**
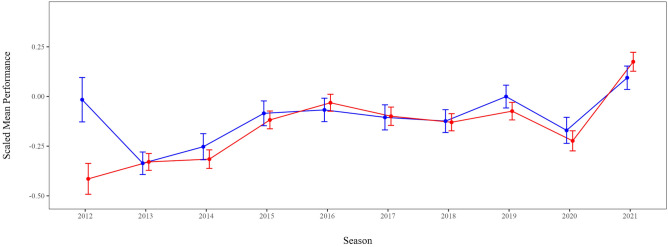
Visualization of the Estimated Means (*EM*) and Standard Errors (*SE*) derived from Model 3 with the predictors season moderated by gender, and age. Male (blue) and female (red) scaled performances developed similarly over the two Olympic cycles. A notable difference is observed in 2012, which is not in line with the other seasons. Again, the scaled performances across all disciplines increased within each Olympic cycle (Rio 2016: 2013–2016; Tokyo 2020: 2017–2021) and peaked in the Olympic year (2016; 2021). The performance decrease in 2020 persisted and was consistent across both genders.

Men and women are expected to differ in their SB per season (average performance), but not in their development across the Olympic cycles. By scaling the performance records per season and gender, the average scaled SB for men and women is 0. Hence, no main effect is analyzed here. Rather, we hypothesized that men and women would differ in their developments over the two Olympic cycles. Hence, gender was added as a moderator to the growth model, which improved the fit slightly but significantly, −2LL_model 3_ = −19,449.60, χ^2^(23) = 32.28, *p* < 0.001, τ = 0.72, ICC = 48%, *R*^2^_cond._ = 0.51. There was no significant or practical difference between men and women in 2013: men’s SB were practically equal to women’s, *b* = −0.01 [−0.12; 0.11], *p* = 0.928, *d* < 0.01. Figure 3 demonstrates that the difference between men and women remained somewhat stable, ranging from *b*_2021_ = −0.08 [−0.19; 0.04], *d* = 0.02 to *b*_2012_ = 0.40 [0.19; 0.62], *d* = 0.06. In 2012, men’s scaled SB were significantly larger than women’s (*p* = 0.0003). The large performance drop in 2020 is still apparent (Fig. 3: women = 0.18 [0.08; 0.27], men = 0.09 [−0.02; 0.21]), as is the performance increase for both athlete groups (women = 0.11 [0.01; 0.20], men = 0.01 [−0.07; 0.18]).

**Fig. 4:**
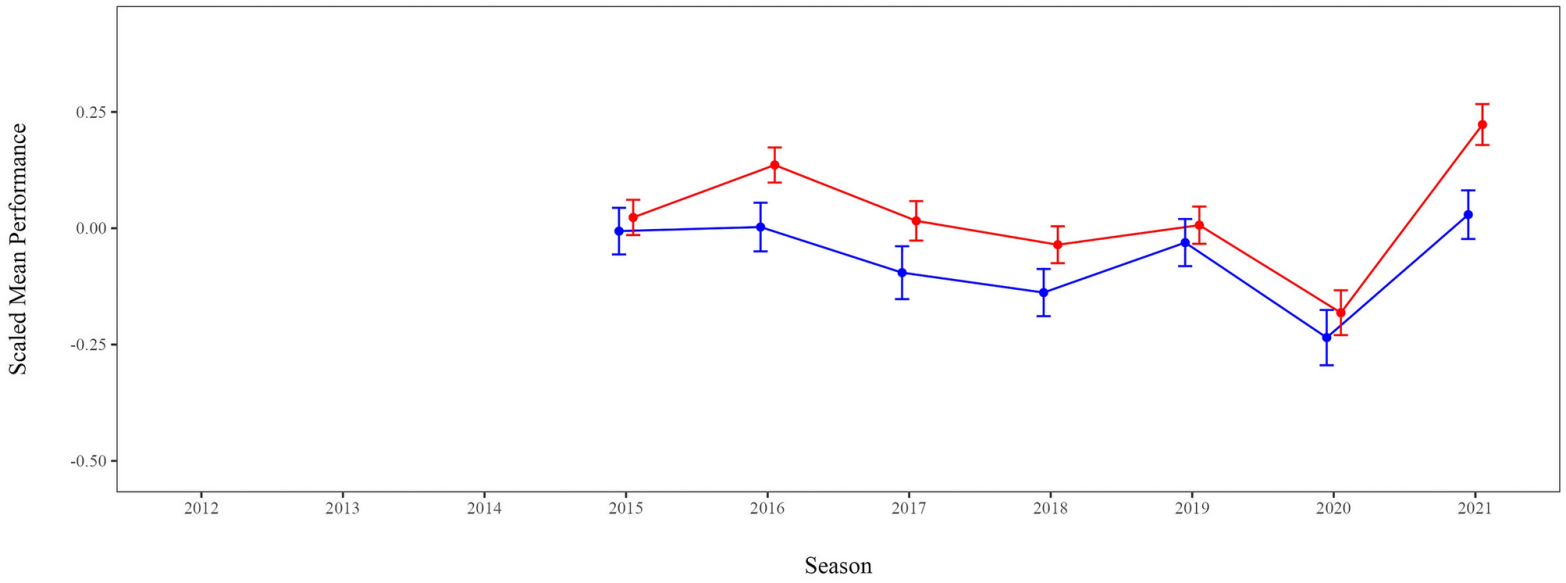
Visualization of the Estimated Means (*EM*) and Standard Errors (*SE*) derived from Model 4 with the predictors season moderated by gender, age and doping affinity. Doping test results were only available since Season 2015. Male (blue) and female (red) scaled performances still developed similarly over the two Olympic cycles but adding doping affinity in comparison to Model 3 highlights that controlling for doping prevalence reduces the effect of season on male performances more compared to female performances. The performance decrease in 2020 persisted and was consistent across both genders.

Lastly, the scaled doping prevalence was added as a predictor. Here, data was only available for the seasons 2015 to 2021, hence, only the complete observations were modeled and no inferential comparison to the other models was possible. With *N* = 4,495, *k* = 868, −2LL_model 4_ = −5,712.11, τ = 0.75, there was still substantial clustering, ICC = 40%, but the additional predictor explained more of the level-1 variance. Doping affinity of the discipline category was not a significant predictor, *b* = 0.01 [−0.05; 0.08], *p* = 0.745, *d* = 0.01. Performance developments were still similar for men and women, *b*_gender_ = 0.01 [−0.15; 0.16], *p* = 0.935, *d* < 0.01 (Fig. 4).

In sum, and to answer the main research question, Fig. 3 displays the estimated means across all disciplines per year and per gender. The results of the final 2-level growth model (Model 3) indicate that age was a relevant linear predictor for performance and that performances developed across disciplines as expected. The official records document a large drop of seasonal bests in 2020, but an even larger improvement in 2021 in both men’s and women’s disciplines.

## Discussion

The results presented here have shed light on the performance development of Olympic track-and-field athletes in the preparation for and the competition at the Olympic Games in Rio in 2016 and Tokyo in 2021. We showed that elite athletes’ performance increases throughout the multi-year cycle to achieve, as planned, maximum performance in the year of the Olympic Games. The 2-level growth model indicated that scaled performance increased significantly from 2013 to 2016 and did not differ in male and female competitors, indicating that they develop similarly over a multi-year cycle. It increased from 2017 to 2019 and dropped significantly in the year of the COVID-19 pandemic, only to recover in 2021 with the highest average performance of all analyzed seasons. We also found that performances after each quadrennial cycle were higher than at the previous cycle, and that the performance progression in the second quadrennial cycle was not as big as in the first.

There are several decreases in performances compared to the previous season (visible in Fig. 3): (a) from 2012 to 2013 (after London 2012); (b) from 2016 to 2017 (after Rio 2016); (c) from 2019 to 2020 (during the COVID-19 pandemic). The performance drop from 2012 to 2013 and the large difference between men and women is not in line with the rest of the aggregated data. This might be explained by the lack of reliable data: the estimated means for the season 2012 are derived from only 209 competition results and 207 different athletes (Table 1). This would negate the unique gender-based difference. The results from other seasons are more in line with previous experimental periodization studies and models^54,55^.

The performance decrease in 2017 relative to 2016 is somewhat expected, since it is the start of a new quadrennial periodization cycle after Rio 2016. Moreover, average age decreased from 2016 to 2017 (Table 1), potentially pointing towards the career end of older athletes or the start or progression of younger athletes. While this difference is negligible (cf. Table 2.), given the elite sample of data here, it is worth investigating more closely to understand the Road to Tokyo 2020 or the successful periodization.

The explanations for the drop in performances in 2020 cannot be confirmed with the present archival data or the existing pool of empirical evidence. A review of the literature emphasizes the disruption of training possibilities and periodization: in the first months of the COVID-19 pandemic, athletes were either unable to train, trained with decreased loads and/or varying degrees of intensity^25^. The recovery of performances in 2021 is therefore somewhat surprising. According to previous studies, the psychological stressors and their potential mitigation through sport psychological consultation appear to enable a supercompensation after the COVID-19 pandemic^30,31^. Moreover, we noted that the athletes that did not continue to compete until Tokyo 2020 were significantly older and are comparable to the athlete drop-outs observed after Rio 2016. Unfortunately, the archival records do not allow for detailed interpretations of age-related career decisions (e.g., injury rates, career transitions, etc.) or the impacts of psychological stressors. These may also be affected by the drastically reduced number of competitions and therefore the limited chance to set high SB that season. Lastly, several athletes might not have competed exhaustively and at their highest capacity in 2020, or they might have extended their off-season preparation. Investigating these reasons quantitively and qualitatively might be interesting for coaches and athletes in the future.

While the ten WRs in 2021 attracted attention, they might not have reflected the performance development of all Olympic athletes. The present analyses and results suggest that on average, an athlete’s performance increased in line with the predicted improvements. The subsample of athletes who did not compete in 2020 and 2021 and who potentially dropped out because of the COVID-19 pandemic, their potential own infection, and the subsequent restrictions on their training and other challenges might not be represented here^26^. Identifying hurdles and reasons for this drop-out in future studies will support career-transitions and federations. For the remaining athletes, the average performance increase might result from successful return to a general preparation period and subsequent progressive loading, extending the macro cycle by one year. Additionally, reduced competition and congested periods in the pre-Olympic year may have reduced injury rates^56^. Some of the improvement might be attributed to measures like home training programs, quarantine training camps and increased investments in psychological support^28,57^.

Due to the limited number of doping controls in 2020^9^, it could be hypothesized that doping had an impact on performance development from 2020 to 2021. However, doping affinity of a discipline category (e.g., sprints, jumps) was not found to explain a significant amount of variance in the present data. Hence, doping affinity does not explain how good or bad performances on average will be. Therefore, the question whether the lack of controls in 2020 really influenced the performance increases from 2020 to 2021 cannot be answered by this analysis of performance records.

### Strengths and limitations

This study possesses several notable strengths that enhance the validity and reliability of its findings. One of these strengths is the 2-level growth model, which considers each athlete individually by modeling their baseline and progression. The ICC (48%) not only justifies but necessitates this approach for future studies that investigate performance developments over multiple seasons. Here, we have controlled for inter-individual differences (gender, age), however, they do not decrease the variability. Especially during the COVID-19 pandemic, clusters in nations or training groups might explain why some athletes continued to perform well and progressed, while others did not compete (well) in 2020 or 2021^58–60^.

The present analysis is limited due to some data and methodological short-comings. First, we rely on the completeness of the performance records at worldathletics.com. However, the number of competitions and athlete records in the seasons prior to 2015 is smaller than in the years 2015–2021, although they are still considered large enough for reliable estimations. Incomplete competition representation at worldathletics.com might have excluded an athlete’s SB. Given the full schedule of Olympic athletes, we expect SB to be achieved at selected competitions rather than undocumented meets not included in this sample, though this cannot be ruled out.

Our findings cover all Olympic athletes and their SB and while we model the growth curve for all performances, no definitive conclusions can be drawn about the explanations for the performance decrease in, for example, 2020. It would be interesting to take a closer look at selected athletes with special performance curves by interviewing them or modeling the macro cycles. The archival data does not allow any interpretation of which athletes were affected least or most by the disruption of periodization schedules or the additional psychological stressors or the restricted access to training resources^26^. Even if more personal information was available, no definitive conclusions about the most disruptive factor can be drawn. For this, different coaches’ responses should be analyzed or surveyed regarding periodization, training accessibility, injury risks, psychological stressors and financial stability^24 ^to identify how to support athletes in hardship or challenging situations effectively and efficiently^26^.

### Future research and conclusion

Performance prediction in this field is not yet possible to the tenth of a second in sprinting or a centimeter in jumping or throwing. The study reveals that Olympic track-and-field athletes experienced consistent performance growth throughout each multi-year Olympic cycle, with peak performances achieved during the Olympic years. Despite significant performance drops in 2020 due to the COVID-19 pandemic, athletes recovered strongly in 2021, achieving the highest average performance of the analyzed seasons. Additionally, while male and female athletes displayed similar developmental patterns, performance gains were smaller in the second quadrennial cycle compared to the first, indicating a plateau effect over time. The final model is at least capable of explaining about 50% of the variability in the data by clustering the performances within athletes, which is not fully explained by gender and age differences. Future research should, where possible, include additional level-2 predictors (e.g., performance indicators, developmental potential) as well as discipline- and country-specific level-1 predictors (skill density, federations) and environmental-factor changes^38^ (e.g., wave light technology).

Additionally, future analyses could model the developments of athletes not only across seasons, but within seasons to draw conclusions about the micro-, meso- and macro-periodization successes and the impact of psychological stressors like financial insecurity, mental-health states and life satisfaction^22^. Although such major disruptions to neither the competition nor the training schedule of elite athletes will likely occur soon, it is important to identify causes of performance- and health-related changes, both positive and negative. Working together with sport practitioners could identify underlying mechanisms for the drop and recovery in 2020 and 2021 through interviews with athletes, coaches and support staff as well as analysis of performance indicators other than records^61^. Additionally, future analyses could compare the Olympic cycles to model the expected logarithmic progression of performance throughout the multi-year cycle and establish whether this progression slope is decreasing throughout the decades. The present results suggest that five years led to larger performance increases, a finding that might be interesting especially for youth training and career planning.

Overall, the findings summarized in Fig. 3 showcase a large performance decrease in SB in 2020, but an even larger improvement in 2021 for both male and female competitors. The findings suggest that for the Paris 2024 Olympics, where the preparation cycle is shortened to three years, performance gains might be smaller than those observed after a four-year cycle but could still exceed the levels of 2021. Coaches should adapt their training schedules to account for the shorter three-year Olympic cycle by emphasizing more efficient periodization strategies, focusing on earlier performance peaks while minimizing injury risks. Given the recovery trends observed after 2020, incorporating targeted psychological and physical resilience-building measures could help athletes maintain consistency during future disruptive periods like red zoning or injury periods, breaks in the competition schedule or congested periods. Additionally, monitoring athletes’ progression more frequently can ensure they remain on track for achieving optimal performance at the targeted competition. Overall, this study contributed to our understanding of the impacts of disruptive periods on elite athletes’ performance progression and development, which will help to optimize training and achieve peak performance in the future.

## Supplementary Information

Supplementary Information.

## Data Availability

Detailed competition results were retrieved from publicly accessible databases and are available at www.worldathletics.org/competition/. A subsample of the extracted records, a list of all included competitions and the R script for replication can be found in this OSF repository: https://osf.io/2evxh/.
