# Supplementary material for: COVID-19 affected elite track-and-field athletes’ Olympic preparation before Tokyo 2020 compared to Rio 2016
Source: Sci Rep. 2025 Feb 19;15:6044. doi: 10.1038/s41598-025-86883-2 (PMC11840131; doi:10.1038/s41598-025-86883-2)
Supplement: Supplementary file 1 — Supplementary Information. [file 41598_2025_86883_MOESM1_ESM.docx]

Supplement 1. *Descriptive statistics of each discipline over the years. Descriptive statistics are provided as mean (standard deviation) in seconds for short distances, minutes for middle and long distances, and metres for throws and jumps. WL = world lead, M = male, F = female.*

| Discipline |  | 2012 | 2013 | 2014 | 2015 | 2016 | 2017 | 2018 | 2019 | 2020 | 2021 |
| --- | --- | --- | --- | --- | --- | --- | --- | --- | --- | --- | --- |
| 100m | M | 11.26 (0.29) WL=10.83 | 11.45 (0.41) WL=10.71 | 11.45 (0.36) WL=10.8 | 11.44 (0.44) WL=10.74 | 11.54 (0.5) WL=10.71 | 11.39 (0.45) WL=10.78 | 11.46 (0.41) WL=10.72 | 11.57 (0.42) WL=10.71 | 11.54 (0.26) WL=10.85 | 11.45 (0.47) WL=10.61 |
|  | F | 10.12 (0.2) WL=9.69 | 10.36 (0.34) WL=9.77 | 10.24 (0.2) WL=9.76 | 10.25 (0.33) WL=9.74 | 10.32 (0.32) WL=9.81 | 10.26 (0.32) WL=9.69 | 10.32 (0.29) WL=9.78 | 10.34 (0.27) WL=9.86 | 10.39 (0.24) WL=9.96 | 10.35 (0.34) WL=9.77 |
| 200m | M | 23.71 (0.67) WL=23.17 | 23.55 (0.79) WL=22.17 | 23.38 (0.86) WL=22.02 | 23.31 (0.93) WL=21.63 | 23.29 (0.86) WL=21.78 | 23.38 (0.87) WL=21.77 | 23.52 (0.86) WL=21.89 | 23.65 (0.92) WL=21.74 | 23.6 (0.58) WL=22.81 | 23.23 (0.86) WL=21.53 |
|  | F | 20.04 (0.46) WL=19.54 | 20.82 (0.66) WL=19.66 | 20.72 (0.53) WL=19.68 | 20.59 (0.52) WL=19.55 | 20.6 (0.47) WL=19.78 | 20.57 (0.54) WL=19.9 | 20.76 (0.61) WL=19.65 | 20.84 (0.64) WL=19.5 | 20.94 (0.54) WL=18.9 | 20.72 (0.55) WL=19.62 |
| 400m | M | 51.2 (1.01) WL=50.03 | 52.49 (1.99) WL=49.33 | 52.48 (1.44) WL=50.12 | 52.03 (1.6) WL=49.26 | 52.25 (1.48) WL=49.44 | 52.49 (1.77) WL=49.46 | 52.98 (1.81) WL=48.97 | 52.92 (1.9) WL=48.14 | 53.19 (1.77) WL=50.98 | 52.18 (1.98) WL=48.36 |
|  | F | 45.07 (0.39) WL=44.37 | 45.79 (1.22) WL=43.74 | 45.94 (1.23) WL=43.74 | 45.26 (1.03) WL=43.48 | 45.98 (1.72) WL=43.03 | 45.58 (1.17) WL=43.62 | 45.74 (1.17) WL=43.61 | 46.3 (1.41) WL=43.48 | 46.67 (1.29) WL=45.05 | 46.02 (1.35) WL=43.85 |
| 800m | M | 2:01.94 (3.03s) WL=1:01.94 | 2:02.22 (3.77s) WL=1:02.22 | 2:02.48 (3.41s) WL=1:02.48 | 2:01.41 (3.5s) WL=1:01.41 | 2:01.86 (3.61s) WL=1:01.86 | 2:01.74 (4.15s) WL=1:01.74 | 2:03.05 (4.17s) WL=1:03.05 | 2:02.94 (4.01s) WL=1:02.94 | 2:03.06 (3.44s) WL=1:03.06 | 2:01.60 (3.66s) WL=1:01.60 |
|  | F | 1:45.35 (2.55s) WL=1:45.35 | 1:47.06 (3.07s) WL=1:47.06 | 1:46.84 (3.25s) WL=1:46.84 | 1:46.70 (2.91s) WL=1:46.70 | 1:47.17 (2.78s) WL=1:47.17 | 1:47.03 (2.56s) WL=1:47.03 | 1:47.13 (2.71s) WL=1:47.13 | 1:47.29 (2.7s) WL=1:47.29 | 1:46.60 (2s) WL=1:46.60 | 1:45.91 (2.05s) WL=1:45.91 |
| 1500m | M | 4:12.91 (8.78s) WL=4:12.91 | 4:10.57 (8.88s) WL=3:10.57 | 4:08.61 (9.01s) WL=3:08.61 | 4:06.75 (7.17s) WL=3:06.75 | 4:09.07 (9.96s) WL=3:09.07 | 4:07.86 (8.31s) WL=3:07.86 | 4:12.28 (9.42s) WL=3:12.28 | 4:11.04 (9.43s) WL=3:11.04 | 4:12.21 (9.32s) WL=3:12.21 | 4:08.65 (8.17s) WL=3:08.65 |
|  | F | 3:35.08 (3.53s) WL=3:35.08 | 3:38.50 (7.79s) WL=3:38.50 | 3:38.96 (7.56s) WL=3:38.96 | 3:39.07 (7.07s) WL=3:39.07 | 3:39.42 (7.16s) WL=3:39.42 | 3:39.13 (6.27s) WL=3:39.13 | 3:40.60 (6.64s) WL=3:40.60 | 3:39.38 (6.14s) WL=3:39.38 | 3:38.37 (6.13s) WL=3:38.37 | 3:37.94 (6.35s) WL=3:37.94 |
| 3000mSC | M | 9:25.59 (10.4s) WL=9:25.59 | 9:41.32 (21.31s) WL=9:41.32 | 9:34.31 (21.03s) WL=9:34.31 | 9:36.40 (16.48s) WL=9:36.40 | 9:36.82 (18.54s) WL=8:36.82 | 9:36.43 (22.07s) WL=8:36.43 | 9:41.74 (23.63s) WL=8:41.74 | 9:41.77 (23.06s) WL=8:41.77 | 9:50.37 (20.59s) WL=9:50.37 | 9:32.68 (19.83s) WL=9:32.68 |
|  | F | 8:22.87 (17.17s) WL=8:22.87 | 8:26.48 (18.06s) WL=7:26.48 | 8:26.29 (14.08s) WL=8:26.29 | 8:29.01 (14.99s) WL=8:29.01 | 8:27.38 (12.32s) WL=8:27.38 | 8:30.63 (14.23s) WL=8:30.63 | 8:31.40 (16.91s) WL=7:31.40 | 8:26.34 (14.27s) WL=8:26.34 | 8:34.01 (16.42s) WL=8:34.01 | 8:22.61 (11.95s) WL=8:22.61 |
| 5000m | M | 14:52.83 (6.68s) WL=14:52.83 | 15:24.24 (48.83s) WL=14:24.24 | 15:22.14 (33.26s) WL=14:22.14 | 15:20.36 (36.54s) WL=14:20.36 | 15:16.03 (34.59s) WL=14:16.03 | 15:04.09 (23.37s) WL=14:04.09 | 15:34.20 (42.35s) WL=14:34.20 | 15:27.23 (41.31s) WL=14:27.23 | 15:35.27 (45.97s) WL=14:35.27 | 15:18.09 (32.08s) WL=14:18.09 |
|  | F | 13:02.67 (4.28s) WL=12:02.67 | 13:22.52 (21.91s) WL=12:22.52 | 13:36.81 (29.61s) WL=12:36.81 | 13:33.32 (32.75s) WL=12:33.32 | 13:33.33 (29.44s) WL=12:33.33 | 13:31.58 (27.4s) WL=12:31.58 | 13:38.99 (31.34s) WL=12:38.99 | 13:36.26 (29.19s) WL=12:36.26 | 13:36.98 (30.56s) WL=12:36.98 | 13:31.83 (29.56s) WL=12:31.83 |
| 10,000m | M |  | 31:47.10 (56.07s) WL=30:47.10 | 32:42.11 (20.62s) WL=32:42.11 | 32:11.31 (29.66s) WL=31:11.31 | 31:32.89 (69.64s) WL=29:32.89 | 31:53.53 (44.71s) WL=30:53.53 | 32:39.84 (64.91s) WL=31:39.84 | 32:13.82 (75.14s) WL=30:13.82 | 33:16.67 (91.33s) WL=30:16.67 | 32:06.36 (92.62s) WL=29:06.36 |
|  | F | 27:07.30 (13.77s) WL=26:07.30 | 27:48.15 (30.39s) WL=27:48.15 | 28:03.92 (59.18s) WL=26:03.92 | 27:52.14 (52.57s) WL=26:52.14 | 27:51.80 (39.67s) WL=26:51.80 | 27:35.90 (47.09s) WL=26:35.90 | 28:28.94 (64.3s) WL=27:28.94 | 28:09.83 (48.57s) WL=26:09.83 | 28:20.74 (52.23s) WL=26:20.74 | 28:08.13 (39.98s) WL=26:08.13 |
| 100mH | F | 13.1 (0.27) WL=12.79 | 13.05 (0.34) WL=12.44 | 13.15 (0.35) WL=12.51 | 13.06 (0.35) WL=12.52 | 12.96 (0.26) WL=12.2 | 13.05 (0.3) WL=12.39 | 13.13 (0.4) WL=12.36 | 13.09 (0.42) WL=12.34 | 13.11 (0.38) WL=12.68 | 12.97 (0.44) WL=12.26 |
|  | M | 13.29 (0.17) WL=13.14 | 13.48 (0.35) WL=13.05 | 13.35 (0.24) WL=12.94 | 13.48 (0.3) WL=12.94 | 13.49 (0.28) WL=12.98 | 13.51 (0.39) WL=13.01 | 13.52 (0.3) WL=12.92 | 13.54 (0.38) WL=12.98 | 13.55 (0.25) WL=13.11 | 13.43 (0.29) WL=12.81 |
| 400mH | M | 56.07 (1.5) WL=53.96 | 56.3 (1.9) WL=52.83 | 57.12 (2.01) WL=54.39 | 56.7 (2.18) WL=53.5 | 56.89 (2.45) WL=53.13 | 56.27 (2.06) WL=53.38 | 56.5 (1.99) WL=53.46 | 56.23 (2.24) WL=52.16 | 56.72 (1.81) WL=53.9 | 55.37 (1.74) WL=51.46 |
|  | F | 48.87 (0.77) WL=48.4 | 50.05 (1.56) WL=47.69 | 49.76 (1.3) WL=48.03 | 49.57 (1.28) WL=47.79 | 49.32 (0.95) WL=47.73 | 49.73 (1.11) WL=48.02 | 49.68 (1.16) WL=46.98 | 49.49 (1.07) WL=46.92 | 50.11 (1.1) WL=46.87 | 49.31 (1.35) WL=45.94 |
| Shot put | M | 19.44 (1.11) WL=20.95 | 17.67 (1.57) WL=20.9 | 17.27 (1.74) WL=20.59 | 17.44 (1.58) WL=20.37 | 17.28 (1.61) WL=20.63 | 17.47 (1.3) WL=19.94 | 17.09 (1.93) WL=20.31 | 17.78 (1.22) WL=20.31 | 17.11 (1.85) WL=19.53 | 18.11 (1.52) WL=20.58 |
|  | F | 21.01 (NA) WL=21.01 | 19.29 (1.31) WL=21.73 | 19.61 (1.5) WL=21.55 | 20.03 (1.26) WL=22.56 | 20.15 (1.25) WL=22.52 | 20.52 (1.2) WL=22.47 | 19.78 (1.55) WL=22.6 | 20.23 (1.65) WL=22.91 | 19.86 (1.85) WL=22.74 | 20.75 (1.23) WL=23.37 |
| Discus  throw | M | 60.9 (4.93) WL=68.77 | 58.4 (6.25) WL=68.96 | 59.15 (6.51) WL=71.08 | 58.97 (6.05) WL=69.28 | 58.96 (6.22) WL=70.88 | 59.79 (5.31) WL=70.31 | 58.45 (5.41) WL=71.38 | 59.64 (5.51) WL=69.39 | 57.31 (5.63) WL=65.93 | 60.58 (6.34) WL=70.22 |
|  | F | 63.95 (2.4) WL=66.84 | 61.52 (3.92) WL=69.75 | 62.11 (3.72) WL=68.36 | 61.78 (3.98) WL=67.4 | 62.22 (3.7) WL=68.37 | 62.45 (4.23) WL=69.21 | 62.14 (4.76) WL=69.72 | 62.78 (4.43) WL=71.86 | 61.99 (3.9) WL=71.37 | 63.38 (4.49) WL=71.4 |
| Hammer throw | M |  | 68.9 (4.79) WL=78.46 | 68.28 (5.86) WL=78.76 | 68.79 (5.4) WL=80.85 | 68.1 (5.66) WL=82.29 | 69.57 (4.24) WL=77.9 | 69.28 (5.44) WL=79.59 | 70.01 (5.58) WL=78.24 | 69.09 (3.72) WL=75.23 | 71.03 (4.95) WL=80.31 |
|  | F |  | 73.91 (4.31) WL=81.97 | 73.88 (4.75) WL=82.69 | 73.61 (3.44) WL=80.88 | 73.57 (3.15) WL=80.93 | 73.67 (3.05) WL=79.81 | 74.66 (3.51) WL=81.85 | 75.37 (2.58) WL=80.88 | 74.45 (3.19) WL=80.28 | 76.13 (3.83) WL=82.98 |
| Javelin | M | 62.98 (2.73) WL=67.19 | 58.38 (5.02) WL=67.7 | 59.57 (6.03) WL=67.99 | 60.44 (4) WL=66.13 | 60.54 (3.84) WL=67.11 | 60.56 (5.1) WL=68.43 | 60.11 (5.14) WL=68.92 | 61.58 (4.3) WL=67.83 | 61.1 (4.12) WL=67.29 | 62.22 (3.19) WL=71.4 |
|  | F | 80.73 (3.98) WL=86.98 | 78.79 (6.13) WL=87.68 | 78.25 (6.78) WL=89.21 | 80.82 (5.55) WL=92.72 | 79.86 (5.93) WL=90.3 | 81.13 (5.39) WL=93.9 | 80.52 (5.27) WL=92.7 | 83.2 (4.07) WL=90.61 | 81.48 (5.39) WL=97.76 | 81.78 (5.61) WL=96.29 |
| Long Jump | M | 6.76 (0.09) WL=6.85 | 6.55 (0.37) WL=7.25 | 6.46 (0.34) WL=7.02 | 6.58 (0.34) WL=7.14 | 6.47 (0.35) WL=7.17 | 6.44 (0.36) WL=7.02 | 6.48 (0.32) WL=7.05 | 6.47 (0.38) WL=7.3 | 6.43 (0.36) WL=7.03 | 6.57 (0.39) WL=7.27 |
|  | F | 7.79 (0.3) WL=8.03 | 7.76 (0.36) WL=8.37 | 7.84 (0.36) WL=8.33 | 7.92 (0.34) WL=8.41 | 7.97 (0.3) WL=8.48 | 8.04 (0.26) WL=8.61 | 7.86 (0.44) WL=8.83 | 7.99 (0.34) WL=8.92 | 7.96 (0.3) WL=8.36 | 8 (0.34) WL=8.6 |
| Triple Jump | M | 14.09 (0.51) WL=14.72 | 13.8 (0.61) WL=14.93 | 13.82 (0.57) WL=15.31 | 13.87 (0.64) WL=15.18 | 13.93 (0.61) WL=15.17 | 14.02 (0.47) WL=14.91 | 14.08 (0.48) WL=14.96 | 13.95 (0.68) WL=15.41 | 13.79 (0.47) WL=14.27 | 14.24 (0.56) WL=15.67 |
|  | F | 16.85 (0.43) WL=17.29 | 16.42 (0.72) WL=17.68 | 16.66 (0.5) WL=17.66 | 16.55 (0.6) WL=18.21 | 16.51 (0.55) WL=17.86 | 16.66 (0.66) WL=18.11 | 16.56 (0.64) WL=17.95 | 16.83 (0.59) WL=18.06 | 16.41 (0.7) WL=17.57 | 16.73 (0.68) WL=17.98 |
| High jump | M | 1.86 (0.05) WL=1.94 | 1.87 (0.07) WL=1.98 | 1.9 (0.06) WL=2.01 | 1.9 (0.05) WL=2.01 | 1.9 (0.05) WL=1.98 | 1.91 (0.06) WL=2.06 | 1.9 (0.07) WL=2.04 | 1.89 (0.09) WL=2.06 | 1.88 (0.07) WL=2 | 1.93 (0.06) WL=2.04 |
|  | F | 2.29 (0.06) WL=2.39 | 2.24 (0.09) WL=2.41 | 2.26 (0.09) WL=2.43 | 2.25 (0.08) WL=2.41 | 2.26 (0.06) WL=2.39 | 2.27 (0.06) WL=2.4 | 2.25 (0.07) WL=2.4 | 2.26 (0.06) WL=2.37 | 2.21 (0.07) WL=2.3 | 2.26 (0.07) WL=2.37 |
| Pole Vault | M | 4.47 (0.17) WL=4.7 | 4.34 (0.28) WL=4.83 | 4.4 (0.26) WL=4.8 | 4.46 (0.27) WL=4.9 | 4.52 (0.2) WL=5 | 4.53 (0.24) WL=4.91 | 4.52 (0.25) WL=4.88 | 4.53 (0.26) WL=4.95 | 4.42 (0.23) WL=4.73 | 4.59 (0.18) WL=4.95 |
|  | F | 5.68 (0.14) WL=5.8 | 5.49 (0.24) WL=6.02 | 5.52 (0.23) WL=5.93 | 5.65 (0.26) WL=6.05 | 5.56 (0.19) WL=6.03 | 5.58 (0.23) WL=5.95 | 5.56 (0.22) WL=6.05 | 5.62 (0.25) WL=6.06 | 5.58 (0.23) WL=6.15 | 5.69 (0.18) WL=6.1 |

Supplement 2. *Performance progression per discipline.*

Supplement 2A. *Performance progression of jumping disciplines.*


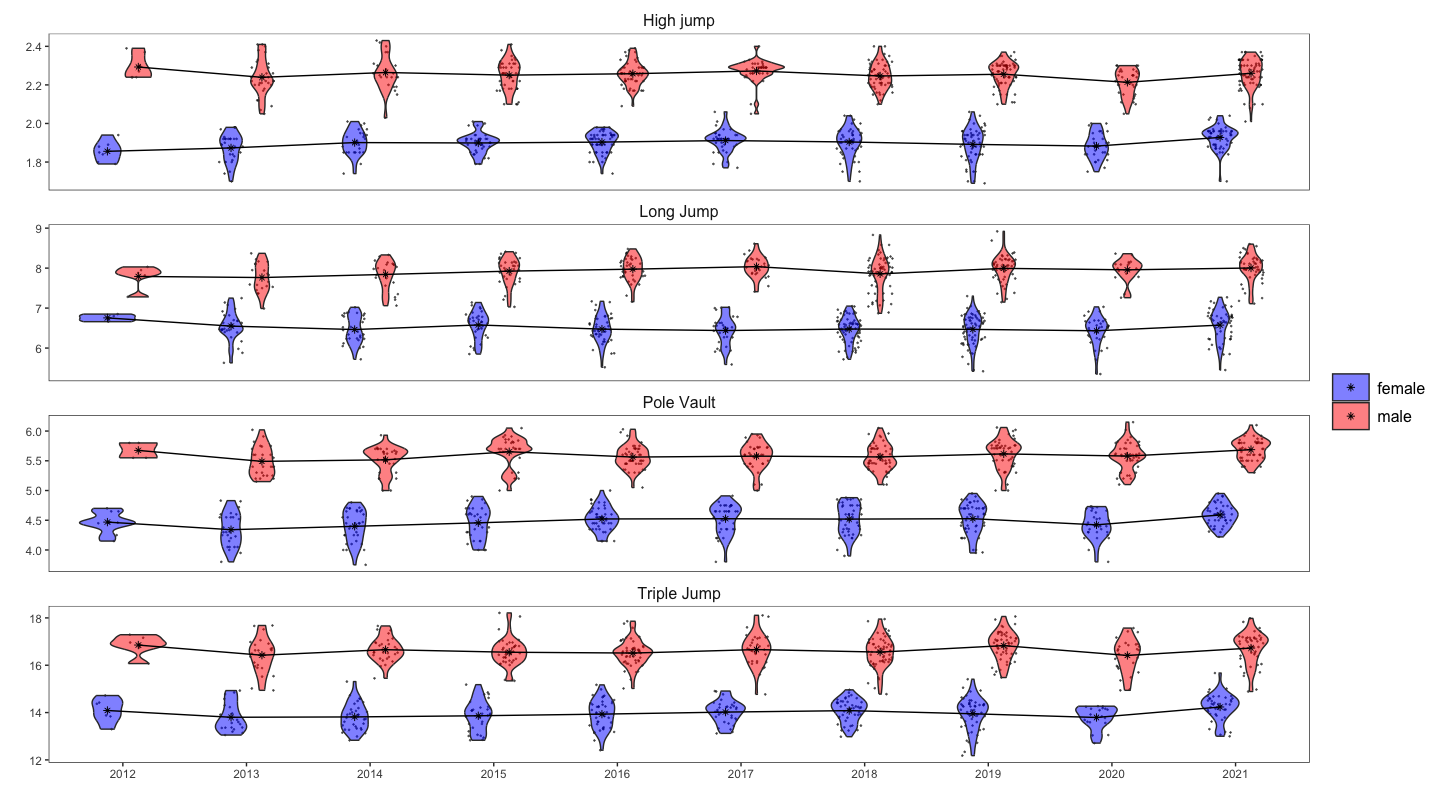


Supplement 2B. *Performance progression of throwing disciplines.*


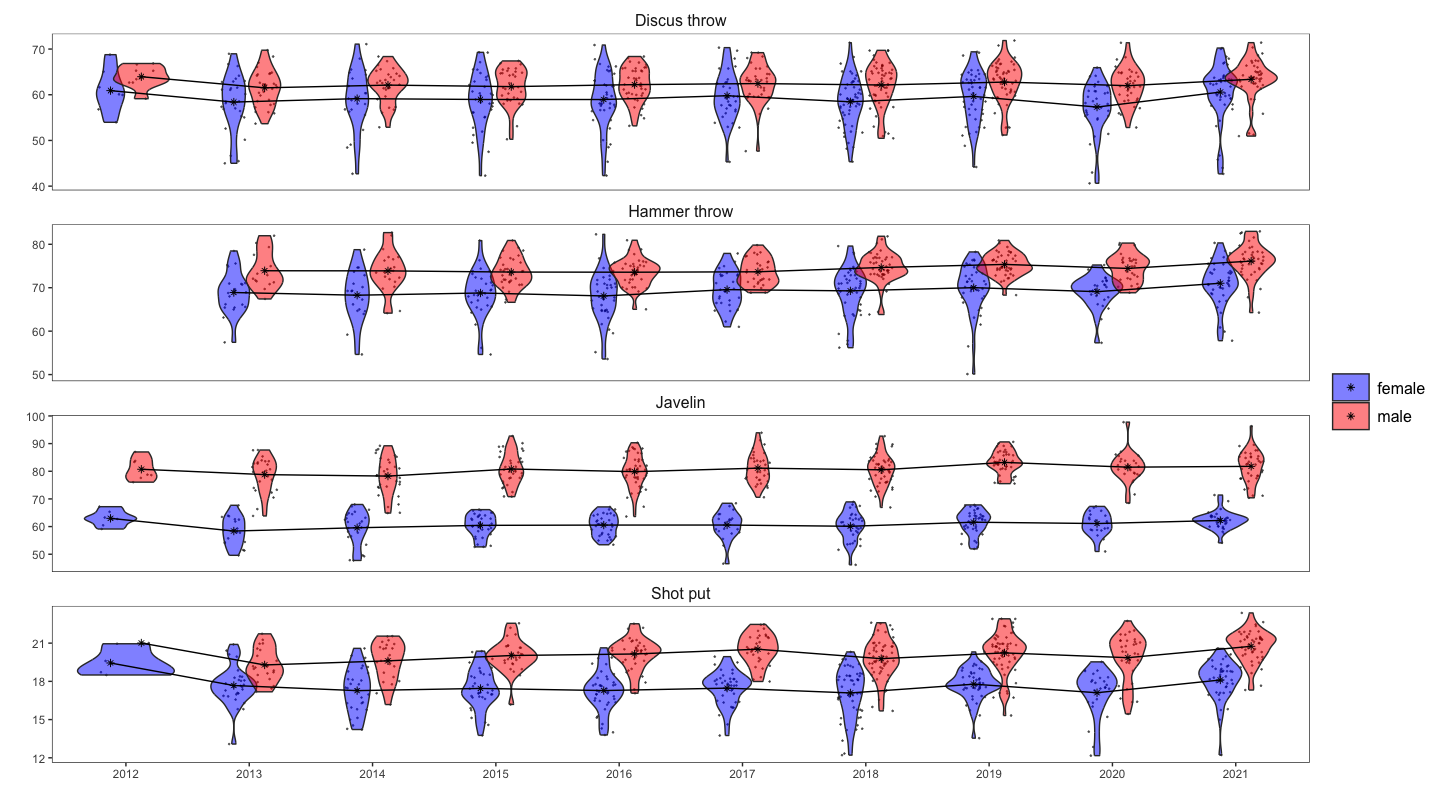


Supplement 2C. *Performance progression of short-distance disciplines.*


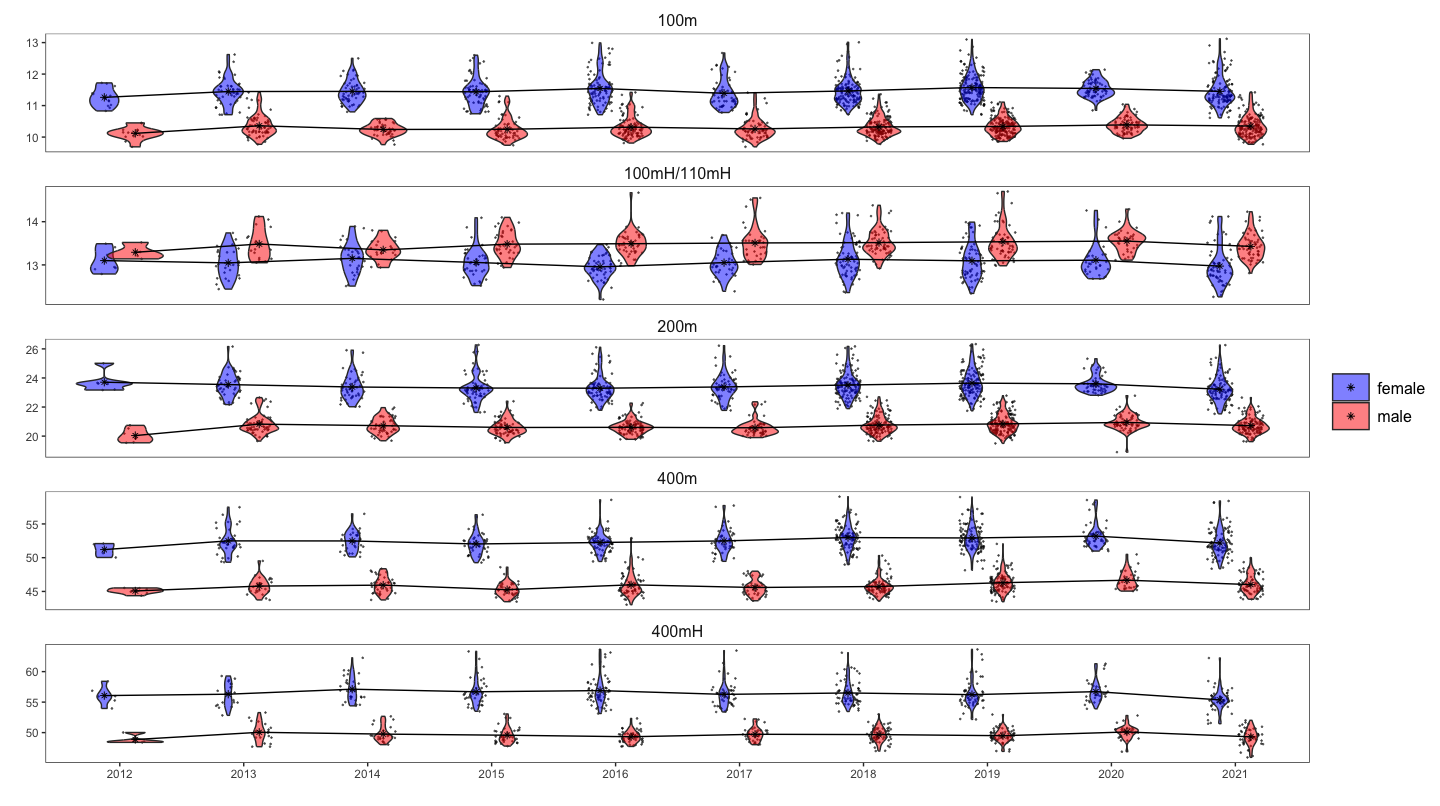


Supplement 2D. *Performance progression of middle-distance disciplines.*


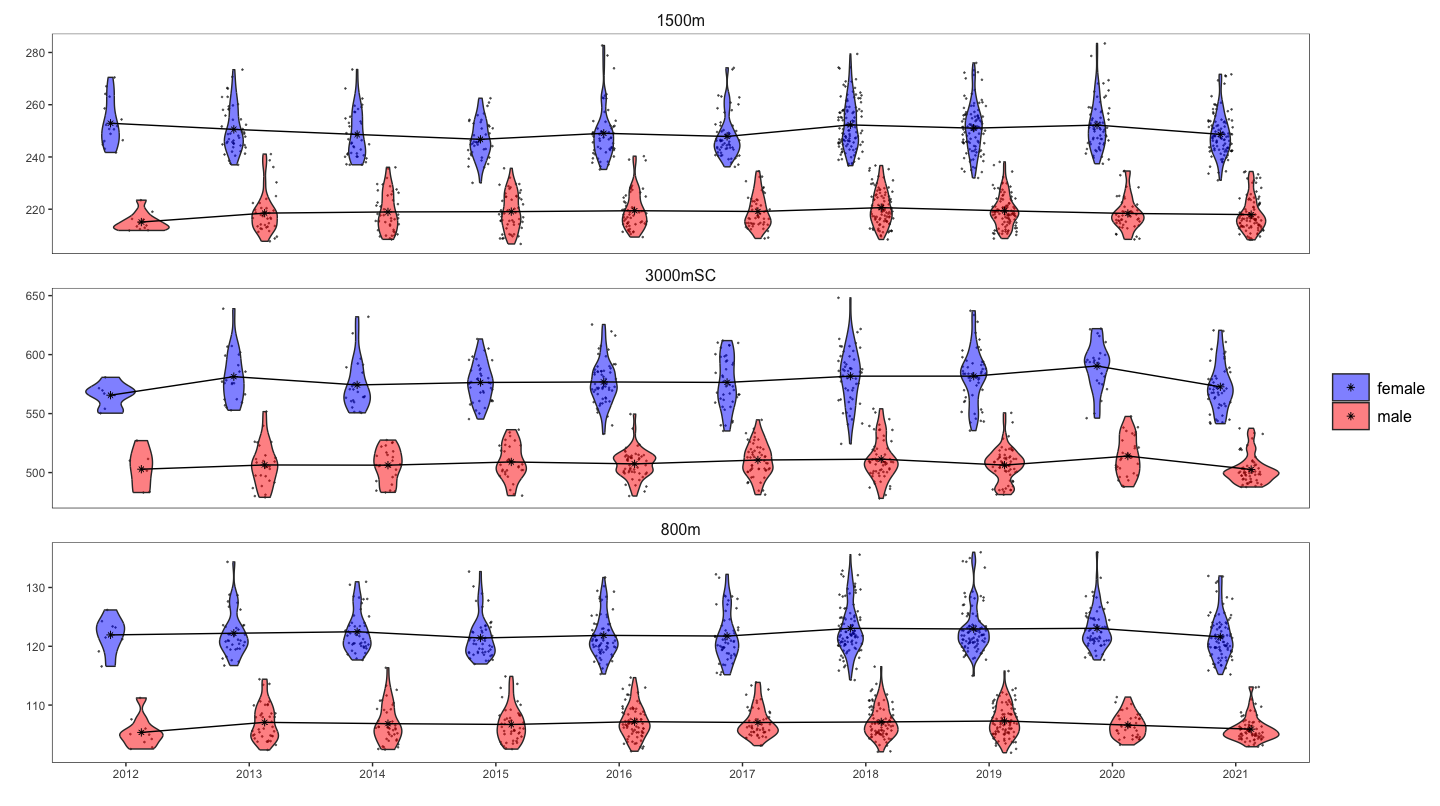


Supplement 2E. *Performance progression of long-distance disciplines.*


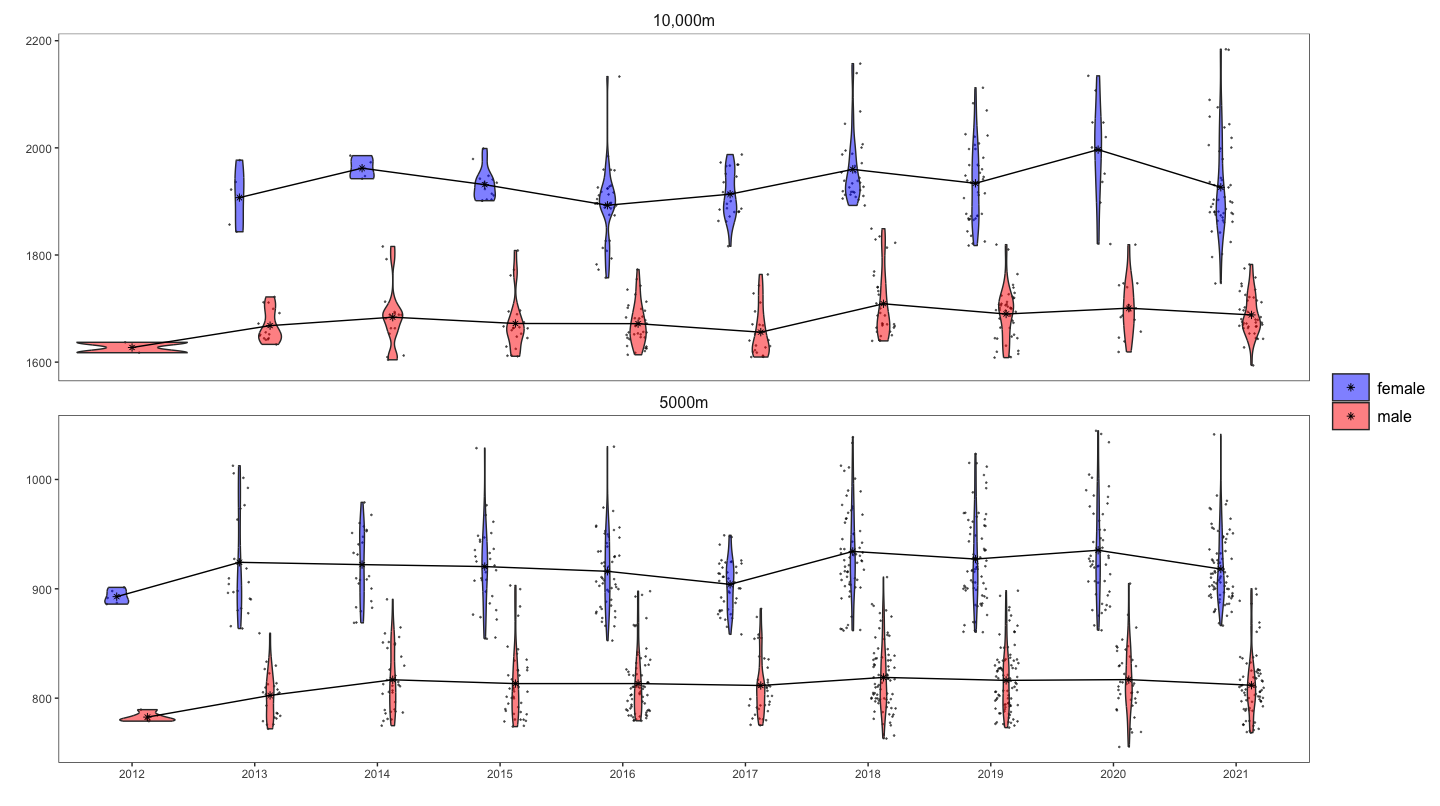


Supplement 3. *Sample statistics for each World Athletics competition category (https://worldathletics.org/world-ranking-rules/basics^33^).*

|  | |  | **Rio 2016** | | | | | **Tokyo 2020** | | | | | |
| --- | --- | --- | --- | --- | --- | --- | --- | --- | --- | --- | --- | --- | --- |
| **Year** |  | | **2012** | **2013** | **2014** | **2015** | **2016** | | **2017** | **2018** | **2019** | **2020** | **2021** |
| OW | *Competitions* | |  | 1 |  | 1 | 1 | | 1 |  | 1 |  | 1 |
|  | *Athletes (% female)* | |  | 321 (47.04%) |  | 596 (50.67%) | 995 (50.95%) | | 564 (46.81%) |  | 256 (53.91%) |  | 508 (48.43%) |
|  | *Performances* | |  | 332 |  | 628 | 1,036 | | 586 |  | 259 |  | 523 |
| DF | *Competitions* | |  |  |  |  |  | | 1 | 2 | 2 | 1 |  |
|  | *Athletes (% female)* | |  |  |  |  |  | | 30 (53.33%) | 26 (34.62%) | 36 (52.78%) | 5 (20.00%) |  |
|  | *Performances* | |  |  |  |  |  | | 30 | 26 | 36 | 5 |  |
| GW | *Competitions* | | 5 | 14 | 15 | 14 | 14 | | 13 | 13 | 11 | 7 | 7 |
|  | *Athletes (% female)* | | 207 (53.14%) | 311 (47.91%) | 450 (46.67%) | 379 (43.27%) | 407 (46.44%) | | 419 (46.54%) | 269 (44.24%) | 229 (39.30%) | 125 (40.00%) | 143 (47.55%) |
|  | *Performances* | | 227 | 338 | 510 | 415 | 446 | | 457 | 296 | 251 | 135 | 151 |
| GL | *Competitions* | |  |  | 1 |  | 1 | |  | 3 | 4 |  | 1 |
|  | *Athletes (% female)* | |  |  | 233 (52.36%) |  | 241 (48.13%) | |  | 219 (45.66%) | 67 (47.76%) |  | 21 (47.62%) |
|  | *Performances* | |  |  | 240 |  | 248 | |  | 228 | 74 |  | 24 |
| A | *Competitions* | |  |  |  |  |  | |  | 11 | 11 | 7 | 8 |
|  | *Athletes (% female)* | |  |  |  |  |  | |  | 323 (49.23%) | 203 (47.78%) | 135 (48.15%) | 111 (34.23%) |
|  | *Performances* | |  |  |  |  |  | |  | 348 | 212 | 141 | 114 |
| B | *Competitions* | |  | 2 | 2 | 2 | 1 | | 1 | 92 | 117 | 78 | 95 |
|  | *Athletes (% female)* | |  | 32 (40.62%) | 87 (58.62%) | 38 (39.47%) | 18 (38.89%) | | 1  (0.00%) | 651 (50.38%) | 699 (49.07%) | 488 (52.87%) | 633 (50.08%) |
|  | *Performances* | |  | 33 | 88 | 39 | 18 | | 1 | 748 | 790 | 582 | 698 |
| C | *Competitions* | |  | 3 | 2 | 2 | 1 | | 5 | 33 | 50 | 17 | 42 |
|  | *Athletes (% female)* | |  | 185 (52.97%) | 160 (48.12%) | 150 (49.33%) | 112 (46.43%) | | 192 (58.33%) | 441 (46.49%) | 368 (49.73%) | 154 (46.75%) | 354 (52.82%) |
|  | *Performances* | |  | 200 | 178 | 164 | 118 | | 210 | 484 | 408 | 163 | 379 |
| D | *Competitions* | |  | 1 | 2 | 2 |  | | 1 | 21 | 42 | 24 | 37 |
|  | *Athletes (% female)* | |  | 85 (56.47%) | 49 (57.14%) | 78 (64.10%) |  | | 58 (50.00%) | 164 (46.95%) | 155 (45.16%) | 134 (49.25%) | 104 (56.73%) |
|  | *Performances* | |  | 92 | 50 | 85 |  | | 61 | 189 | 170 | 142 | 119 |
| E | *Competitions* | |  |  |  | 1 | 1 | | 1 | 21 | 98 | 29 | 68 |
|  | *Athletes (% female)* | |  |  |  | 25 (44.00%) | 23 (52.17%) | | 14 (50.00%) | 128 (55.47%) | 308 (48.05%) | 85 (54.12%) | 196 (42.35%) |
|  | *Performances* | |  |  |  | 31 | 25 | | 18 | 138 | 352 | 90 | 215 |
| F | *Competitions* | |  |  |  |  |  | |  |  | 1 |  | 1 |
|  | *Athletes (% female)* | |  |  |  |  |  | |  |  | 10  (30.00%) |  | 2  (50.00%) |
|  | *Performances* | |  |  |  |  |  | |  |  | 10 |  | 2 |
| Category “OW” = strongest competitions (e.g., World Championships, Olympic Games), DF = Diamond League Finals, GW = Gold World Athletics competitions, Diamond League Meetings and equivalents, GL = Area Senior Outdoor Championships. Other competitions and meetings are classified in A to F. | | | | | | | | | | | | | |
